# Bio-physically plausible visualization of highly scattering fluorescent neocortical models for in silico experimentation

**DOI:** 10.1186/s12859-016-1444-4

**Published:** 2017-02-15

**Authors:** Marwan Abdellah, Ahmet Bilgili, Stefan Eilemann, Julian Shillcock, Henry Markram, Felix Schürmann

**Affiliations:** Blue Brain Project (BBP), École Polytechnique Fédérale de Lausanne (EPFL), Biotech Campus, Chemin des Mines 9, Geneva, 1202 Switzerland

**Keywords:** Modeling and simulation, Highly scattering volumes, Fluorescence rendering and visualization, Neocortical brain models, In silico neuroscience, Modelling and Simulation

## Abstract

**Background:**

We present a visualization pipeline capable of accurate rendering of highly scattering fluorescent neocortical neuronal models. The pipeline is mainly developed to serve the computational neurobiology community. It allows the scientists to visualize the results of their virtual experiments that are performed in computer simulations, or *in silico*. The impact of the presented pipeline opens novel avenues for assisting the neuroscientists to build biologically accurate models of the brain. These models result from computer simulations of physical experiments that use fluorescence imaging to understand the structural and functional aspects of the brain. Due to the limited capabilities of the current visualization workflows to handle fluorescent volumetric datasets, we propose a physically-based optical model that can accurately simulate light interaction with fluorescent-tagged scattering media based on the basic principles of geometric optics and Monte Carlo path tracing. We also develop an automated and efficient framework for generating dense fluorescent tissue blocks from a neocortical column model that is composed of approximately 31000 neurons.

**Results:**

Our pipeline is used to visualize a virtual fluorescent tissue block of 50 *μ*m^3^ that is reconstructed from the somatosensory cortex of juvenile rat. The fluorescence optical model is qualitatively analyzed and validated against experimental emission spectra of different fluorescent dyes from the Alexa Fluor family.

**Conclusion:**

We discussed a scientific visualization pipeline for creating images of synthetic neocortical neuronal models that are tagged virtually with fluorescent labels on a physically-plausible basis. The pipeline is applied to analyze and validate simulation data generated from neuroscientific in silico experiments.

## Background

Scientific visualization is a key component in neurobiology. It helps neurobiologists to explore and convey different levels of interpretations of complex sets of neuroscientific data. Recent advances in computational sciences and hardware technologies allowed some biological experiments to move from the wet laboratory to computer simulations, to *in silico* [1–3] experiments.

This paradigm shift is expected to accelerate and consolidate the research discovery and also to enable novel capabilities in the near future. It will reduce the dramatic costs of clinical trials and complement the traditional in vivo and in vitro methods [4, 5]. Nevertheless, this approach requires developing rigorous mathematical models of the biological experiments and their surrounding physical conditions and then plugging them in high performance computer simulation applications. These applications are designed to exploit the growing computing power of state-of-the-art supercomputers to simulate and analyze complex biological processes at different scales of resolution [6].

This emerging trend opens novel avenues for multi-scale computational modeling of the brain tissue, and in turn a better understanding of how the brain works.

In this context, visualization is not merely exploited for providing visual analysis of the data; it is a significant tool for evaluating and validating the results of in silico experiments. This visual feedback closes the loop and affords the neuroscientists an effective environment to tune and enhance their models and also to improve the accuracy of the simulations in an iterative manner.

### Motivation

The current neuroscientific visualization tools have been improved considerably during the last years to visualize simulation data. A clear example is given by Hernando et al. to interactively visualize the simulation of the cortical activity of large scale neuronal microcircuits [7]. Nevertheless, the toolset is still inadequate for visualizing and validating the data generated from various in silico experiments such as voltage sensitive dye imaging (VSDI) [8], Calcium imaging [9] and also optogenetic stimulation experiments. For example, visualizing the data arising from simulating an optogenetic procedure entails incorporating plausible optical models into the visualization pipeline to account for light interaction with highly scattering turbid media [10]. Accurate visualization of the responses from simulated imaging experiments requires a sophisticated bio-physically-based optical model that incorporates fluorescence in the rendering integral and can account for the actual optical properties of the biological tissue. Such pipeline is still largely unfulfilled and will require an extensible spectral visualization system that can model and simulate light interaction with highly scattering fluorescent volumetric data resembling the fluorescent structures in real tissue.

We address these shortcomings and present an advanced visualization pipeline that can accurately render highly scattering fluorescent volumetric datasets. This pipeline is mainly applied to a fluorescent brain model that represents a digital reconstruction of the microcircuitry of somatosensory cortex of rats [9] to validate its structural and functional aspects. For instance, it is currently used to perform in silico VSDI experiments for validating the cortical activity of the reconstructed model against in vivo imaging experiments [8]. Moreover, it can be useful for other fields such as computational microscopy, where a physically-plausible simulation of microscopic fluorescent images is required for analysis purposes [11–13].

Our pipeline is composed of two software workflows. The first one is a generic physically-based visualization engine for rendering highly scattering heterogeneous fluorescent volumes. The other workflow is developed in particular to efficiently extract a fluorescent tissue block volume from the neocortical column micro-circuit presented by Markram et al. [9].

### Contributions

1. Design and implementation of an extensible pipeline for visualizing fluorescent-tagged scattering volumetric datasets.
2. Rigorous physically-based optical model to simulate the light interaction with fluorescent participating media, taking into account their spectroscopic and optical properties.
3. Qualitative validation and analysis of the developed optical model by correlating the spectral power distributions (SPDs) (or responses) of the generated images with respect to experimental emission spectra of different fluorescent dyes.
4. Design and implementation of an automated parallel workflow for generating an extracted fluorescent tissue block from the neocortical column model.
5. Visualization of fluorescent neuronal models tagged with multiple fluorescent solutions having different optical properties and evaluating the results collaboratively with neurobiologists.

### Related work

Neurobiology scientists are familiar with generic visualization packages such as Paraview [14], Voreen [15] and ImageVis3D [16]. They use them frequently to visualize and analyse data acquired from sensing devices, for example imaging scanners and microscopes. In some cases, these software packages can be employed for visualizing certain *structural* aspects of the data arising from in silico experiments and modeling procedures, for example, to validate the morphological distribution of the neurons in the neocortical column model [9]. Other frameworks have been customized to fulfill specific demands required by the scientists such as Voxx [17] and VAA3D [18]. The design goals of the previous frameworks have been focused on scalability and interactivity. Consequently, they traded the performance with oversimplified optical models that remain very limited to visualize fluorescent data or even to enhance the photorealism of the generated image [19, 20].

Photorealistic visualization of neuroscientific data with advanced illumination models was addressed in two studies. The first one is presented by Banks et al. [21]. They integrated global illumination into their visual data analysis pipeline for displaying the fiber tracts of the brain. Their study was intended to improve the data interpretation in the presence of complex jungle of fibers surrounding brain tumors. The other study presented Exposure render, an interactive GPU-based framework that coupled Monte Carlo ray tracing with physically-based light transport models to generate highly realistic renderings of volumetric data [22]. This framework is capable of visualizing in silico optogenetic experiments, but it cannot be employed to visualize fluorescent data.

Visualizing fluorescent volumetric data was firstly presented in FluVR [23], a commercial application that used a simple deterministic physically-based model called the simulated fluorescence process (SFP) to combine elastic and inelastic rendering. Although it was capable of handling multiple fluorescent dyes in the volume, FluVR was limited in several regards. The SFP assumed that the emission occurs only at the maximum emission wavelength and ignored the rest of the emission spectrum. This optical model did not account for the spectral characteristic of the dyes and ignored multiple scattering.

Physically-plausible visualization of fluorescent participating media has been investigated in few computer graphics research studies. These studies were exclusive to specific applications and their implementations were not developed in the form of an integrated framework that could be utilized for other purposes. In summary, these studies have developed extensions to integrate the fluorescence phenomena into the rendering equation [24–28], but they were limited to certain extent. Glassner [24] presented the first formalism of the full rendering equation to simulate the fluorescence effect. However, his model ignored the distinct properties of the fluorescent dyes. Cerezo [27, 28] and Gutierrez [25, 26] have extended Glassner’s model to account for these missing parameters. Nevertheless, their models used biased rendering methods (discrete ordinates and curved photon mapping) to render the fluorescent pigments of the ocean. Moreover, they ignored the actual spectral properties of the dyes and used oversimplified profiles for the excitation and the emission spectra. Abdellah et al. presented a physically-based framework for simulating imaging experiments with light sheet fluorescence microscopy. The optical model developed in this study presented further extension to the previous fluorescence models taking into account the intrinsic characteristics of fluorescent dyes [29, 30]. They also validated their model against realistic emission spectra of multiple fluorescent dyes. This model was only capable of visualizing tissue models with negligible scattering properties to simulate the imaging of clarified brain tissue [31, 32], but it failed to handle volumetric tissue models with highly scattering content. Our optical model presented in the following section is introduced to fill this gap.

## Methods

### Optical models

Based on ray tracing and the basic principles of geometric optics, advanced optical models of volume rendering ideally solve the radiative transfer equation (RTE) to simulate the light transport in a continuum and generate a physically-plausible synthetic image [33–35]. The general formulation of the light transport presented by Veach [36] is extended by Pauly et al. [37] to handle scattering media. Nevertheless, this formulation has never been investigated for considering the fluorescence effects. In the following part, we begin with this extension to derive the path integral formulation of our fluorescence optical model. Table 1 summarizes all the relevant terms and symbols that appear later in the text. We also recommend the reader to refer to [38] for further explanation of some of the terms in the rendering integrals.

**Table 1:** Summary for all the symbols that are used in the text

*λ*	Wavelength
*λ*_*x*_	Excitation wavelength
*λ*_*m*_	Emission wavelength
*ϕ*	Quantum yield
*ε*	Molar absorptivity
*M*	Molecular weight
*f*_*x*_	Fluorophore excitation spectrum
*f*_*m*_	Fluorophore emission spectrum
*L*_ve_	Self-emission radiance at point p
*L*_*s*_	Radiance due to scattering
*L*_*i*_	Incoming radiance to point p
*L*_e_	Outgoing radiance emitted from the light source
*x*_*n*_	Point on the light source for a path consisting of *n* points
*x*_0_	Point on the surface of the virtual film of the camera
*x*_*i*_/*x*_*j*_	Point along the path after *i* or *j* bounces
$\overline {x}$	Path connecting the camera and light source
*C*	Concentration
*ω*	Direction
*ω* ^′^	Incoming direction
*F*_*s*_	Scattering function
*G*	Geometry term
*V*	Binary Visibility
$V_{f_{i}}$	Path Binary Fluorescence Visibility
*τ*	Transmittance
$\hat {V}$	Visibility term
*σ*_s_	Scattering coefficient
*σ*	Absorption coefficient
*f*_*p*_	Phase function
*p*_*x*_	Photon excitation (or absorption) probability
*p*_*m*_	Photon emission probability

### Path integral formulation in fluorescent volumes

**Fig. 1:**
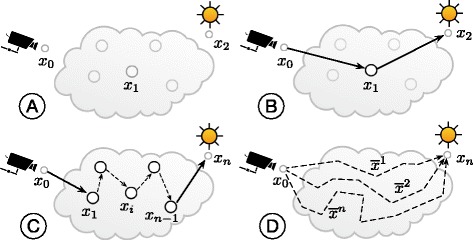
Light transport in a highly scattering volumetric extent. **a** The volume prior to illumination by the light source. **b** Single scattering interaction: the light ray is scattered once between the light source and the camera on a single path *x*_0_ *x*_1_ *x*_2_. **c** Multiple scattering: the light ray bounces multiple times between several interaction events before reaching the camera on a single path *x*_0_ *x*_1_ *x*_2_…*x*_*n*−1_ *x*_*n*_. **d** The radiative transport equation evaluates the light propagating from the light source to the camera on multiple paths $\overline {x}^{1}, \overline {x}^{2}, \ldots, \overline {x}^{n}$. The rays are shot from the camera towards the light source to sample the scattering events

Assuming a path consisting of three points *x*_0_ *x*_1_ *x*_2_, where the light source and the camera film are located at points *x*_0_ and *x*_2_ respectively, and *x*_1_ is sampled to be a random interaction point in the volume where the light scattering occurs (Fig. 1), the radiance arriving to the camera following a scattering event at *x*_1_ can be computed with the monochromatic light transport formula described in Eq. (1), where *ω*=*x*_0_←*x*_1_ is the incoming direction, *L*_ve_ and *L*_*s*_ are the radiance due to self emission and scattering respectively. The self-emission term is usually ignored unless the volume itself is emitting due to chemical or thermal processes, which is out of the scope of the presented model. In this case, the total radiance recorded by the camera due to light scattering *L*_s_ in the volume is evaluated with the integral in Eq. (2), where *σ*_*s*_ and *f*_*p*_ are the scattering coefficient and the phase function of the volume respectively and *L*_*i*_ is the incoming radiance towards the point *x*_1_ from any direction *ω* ^′^.

1 $$  \begin{aligned} L&(x_{0}, \omega) = \\ L(x_{0} \leftarrow x_{1}) = L_{\text{ve}}&(x_{0} \leftarrow x_{1}) + L_{\mathrm{s}}(x_{0} \leftarrow x_{1}) \end{aligned}  $$

2 $$  \begin{aligned} L&(x_{0} \leftarrow x_{1}) \Big|_{\mathrm{No \, Self \, Emission}} = \\ \sigma_{s}(x_{1}, x_{0} \leftarrow x_{1})& \int_{\Omega_{4\pi}} f_{p}(x_{1}, x_{0} \leftarrow x_{1}, \omega')~~ L_{i}(x_{1}, \omega') \, \mathrm{d}\omega' \end{aligned}  $$

For convenience [39], Eq. (2) can be re-written in the form of Eq. (3) as an integral over surfaces d*A* and volumes d*V* instead of directions d*ω* ^′^ on the sphere *Ω*_4*π*_ to yield what is called the *three-point form* of the light transport equation, where *F*_s_, *G*, $\hat {V}$, *V*, *τ* and *L*_*e*_ are the scattering function, geometric term, visibility term, binary visibility function, transmittance and the emitted radiance from the light source at *x*_2_ respectively.

3 $$  \begin{aligned} L_{\mathrm{s}}(x_{0} \leftarrow x_{1}) &= \int_{A} L_{\mathrm{e}}(x_{1} \leftarrow x_{2}) \\ F_{\mathrm{s}}(x_{0} \leftarrow x_{1} \leftarrow x_{2}) ~~ &G(x_{2}, x_{1}) ~~ \hat{V}(x_{2}, x_{1}) ~~ \mathrm{d}{A(x_{2})} \end{aligned}  $$

where



If the light scatters at *n*−1 interaction sites before reaching the camera at *x*_0_, where *x*_*n*_ is a sampled point on the light source, the path integral equation becomes

8 $$  \begin{aligned} &\qquad\qquad\qquad\qquad L(x_{0}, \omega) = \\ &\overbrace{\int_{A} \ldots \int_{V}}^{n - 1} L_{{e}}(x_{n - 1} \leftarrow x_{n}) G(x_{n - 1}, x_{n}) \hat{V}(x_{n - 1}, x_{n}) \\ &\prod_{i = 1}^{n - 1} \left[ F_{\mathrm{s}}(x_{i + 1} \leftarrow x{i} \leftarrow x_{i - 1}) G(x_{i + 1}, x_{i}) V(x_{i +1}, x_{i}) \right] \\ &\qquad\qquad\qquad\qquad \mathrm{d}V(x_{{1}}) \ldots \mathrm{d}A(x_{{n}}) \end{aligned}  $$

where *L*_*e*_ is the emitted radiance from the light source at the sampled point on its surface *x*_*n*_ to the first interaction point in the volume *x*_*n*−1_.

In principle, Eq. (8) can be used to render highly scattering volumetric models assuming monochromatic wavelengths, i.e. there is no transfer of energy from one wavelength to another. We have extended this equation by introducing a term called the *path binary fluorescent visibility* $V_{f_{i}}$ that indicates whether a path has encountered a fluorescence emission or not. Adding this term to Eq. (8) and integrating over all excitation wavelengths *λ*_*x*_ to evaluate the radiance at specific emission wavelength *λ*_*m*_, the rendering equation becomes

9 $${}  \begin{aligned} &L(x_{0}, \omega, \lambda_{m}) = \int_{\lambda_{x}} \overbrace{\int_{A} \ldots \int_{V}}^{n - 1} L_{{e}}(x_{n - 1} \leftarrow x_{n}, \lambda_{x}) \\ &\quad G(x_{n}, x_{n - 1}) \hat{V}(x_{n}, x_{n - 1}, \lambda_{x}) V_{f_{i}}(\lambda_{x}, \lambda_{m}) \times \\ &\prod_{i = 1}^{n - 1} \left[ F_{\mathrm{s}}(x_{i + 1} \leftarrow x_{i} \leftarrow x_{i - 1}, \lambda_{m}) G(x_{i + 1}, x_{i}) \ V(x_{i +1}, x_{i}) \right] \\ &~~~~~~~~~~~~~~~~~~~~~~~~~~~~~~~\mathrm{d}V(x_{{1}}) \ldots \mathrm{d}A(x_{{n}}) \mathrm{d}\lambda_{x} \end{aligned}  $$

### Monte Carlo estimator

The path integral formulation of our fluorescence model, Eq. (9), evaluates the radiance arriving to the camera at point *x*_0_ from direction *ω* at certain emission wavelength *λ*_*m*_ after multiple scattering events in a highly scattering fluorescent volume. In a stochastic path tracer, this integral can be approximated with the Monte Carlo estimator expressed by Eq. (10), where *p*(.) is the probability density function (PDF) for sampling a point *x*_*n*_ on the surface of the light source, an excitation wavelength *λ*_*x*_ from the emission spectrum of illuminating light, a scattering event with a direction *ω*_*j*_ and a distance *t*_*j*_. The path binary fluorescence visibility term $V_{f_{i}}$ accounts for the spectral optical properties of the volume, the intrinsic spectroscopic properties of the fluorescent dye including its excitation and emission spectra, molar absorptivity and quantum yield, and also the concentration of the fluorescent solvent in a given solution.

10 $$  \begin{aligned} &\qquad\qquad\qquad L_{\mathrm{i}}({x}_{0}, \omega, \lambda_{{m}}) \approx \\ &\frac{1}{N_{\lambda}} \frac{1}{N} \sum_{\lambda = 1}^{N_{\lambda}} \sum_{i = 1}^{N} \frac{L_{\mathrm{e}}({x}_{n},\lambda_{\mathrm{x}}) \hat{V_{i}}}{p({x}_{n}) p(\lambda_{{x}})} V_{{f}_{i}} \prod_{j = 1}^{M} \frac{\hat{V_{j}} {F_{j}} G_{j}}{p(\omega_{j}) p(t_{j})} \end{aligned}  $$

where 

**Fig. 2:**
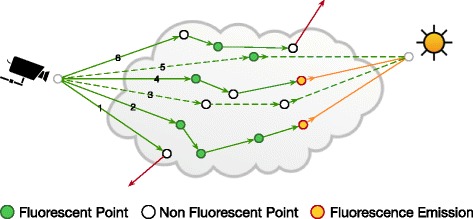
Path tracing with multiple scattering in fluorescent volume. The *green* and *yellow* rays are transported at *λ*_*m*_ and *λ*_*x*_ respectively. The *red* rays escape the volume with no contribution to the estimated radiance along the path. The *dashed* rays indicate invalid paths, where fluorescence visibility is zero. The light is only sampled if a fluorescence emission event is determined

**Fig. 3:**
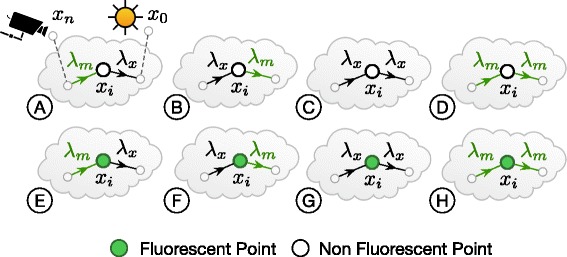
All possible combinations of interaction events during path sampling in a scattering fluorescent mixture. The white/green events represent an interaction between the light ray and non-fluorescent/fluorescent volume samples. The events in (**a**) and (**b**) are not physically-plausible because a fluorescent emission must occur at a fluorescent sample. **f** is also not possible because *λ*_*m*_ cannot excite the dyes to emit at *λ*_*x*_. The events in (**c**), (**d**), (**g**) and (**h**) represent an elastic scattering at the same wavelength. **e** is the only event that can account for fluorescence emission

Monte Carlo path tracing is used to determine the interaction sites, or *events*, within the volume extent. The fluorescent events – represented by the green points in Fig. 2 – are stochastically identified according to the ratio between the fluorescence absorption coefficient ${\mu _{a}^{f}}$ and the total absorption coefficient *μ*_*a*_ of the volume at emission wavelength *λ*_*m*_. There are eight possible combinations that might occur during the path sampling. According to the type of the sampled event, some of these cases are plausible and the other are not possible as explained in Fig. 3. The SPD of the fluorescence absorption coefficient ${\mu _{a}^{f}}(\lambda)$ is expressed in terms of the excitation (or absorption) spectrum of the fluorophore *f*_*x*_(*λ*), the concentration of the dye in the solution *C*, and its molar absorptivity at the maximum excitation wavelength *ε*. The spectral radiance is computed by tracing a ray through the volume at certain wavelength between 300 and 800 nm with 1 nm increments. The estimated pixel value is updated only if the constructed path is valid and a fluorescence emission occurs. A valid contributing path, such as 2 and 4 in Fig. 2, consists of a series of elastic scattering events and a single inelastic one that involves changing the wavelength from *λ*_*x*_ to *λ*_*m*_. In this case, the light source is sampled and the radiance emitted towards the fluorescence emission event is attenuated according to *λ*_*x*_. Otherwise, the fluorescence visibility *V*_*f*_ term is set to zero and the path is terminated. The paths are sampled with woodcock tracking, which is known to be an unbiased method [40, 41].

The probability of fluorescence emission *p*_*f*_ is expressed by two terms: the photon absorption probability *p*_*x*_ and the photon emission probability *p*_*m*_ [42], i.e. *p*_*f*_(*λ*_*x*_,*λ*_*m*_)=*p*_*x*_(*λ*_*x*_)*p*_*m*_(*λ*_*m*_) where

15 $$  p_{{x}}(\lambda_{{x}}) = \phi \frac{{\mu_{a}^{f}}(\lambda_{{x}})}{\mu_{a}(\lambda_{{x}})}  $$

16 $$  p_{{m}}(\lambda_{{m}}) = \frac{f_{{{m}}}(\lambda_{{m}}) \Delta \lambda}{\displaystyle \int_{0}^{\infty} f_{{{m}}}(\lambda) \mathrm{d}\lambda}  $$

Therefore, the fluorescence emission probabilistically occurs in terms of the exact spectral characteristics of the fluorescent dye including its excitation *f*_*x*_(*λ*) and emission *f*_*m*_(*λ*) spectra, and its quantum yield *ϕ*. This method can accurately generate fluorescent images with SPDs that have similar profiles to the actual emission spectra of the fluorescent dyes. Though, it ignores secondary fluorescence effects such as quenching, photo-bleaching or saturation.

### Virtual fluorescent tissue volume generation

**Fig. 4:**
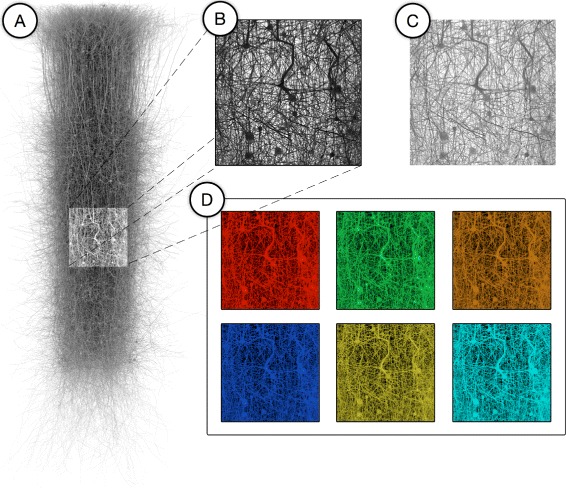
The process of creating a fluorescent tissue block from the cortical column model. **a** The meshes of each neuron in the column are created and loaded according to their position and orientation specified by a given micro-circuit configuration. **b** The requested mesh block is extracted from the neocortical column model in (**a**). **c** The mesh block is converted into a volume with solid voxelization. **d** The volume block is annotated with the optical properties of the brain and the spectroscopic properties of the dyes specified in the input configuration file. The density of the cells in the illustrated model in A is only 5*%*

The digital model of the neocortical column is organized in a *circuit*, which can be seen as a database containing a set of neurons having diverse morphological and electrical characteristics. These neurons are statistically positioned and oriented within the 3D extent of the column [9, 43]. The virtual fluorescent tissue block is reconstructed from the neocortical column circuit for in silico experiments in five basic steps (Fig. 4):

1. Identifying a list of neurons that will be contained in the resulting tissue block. This list can be selected based on common morphological or electrical properties to address specific kind of in silico experiment.
2. Creating a *watertight* surface mesh model for the block from the morphological descriptions of the neurons in the circuit. If a given morphology is broken, the neuron identifier is reported to fix the morphology. The neuronal morphologies are converted into watertight surface meshes using an extended version of the workflow presented by Lassare et al. [44]. The individual meshes generated for every neuron are loaded into Blender [45] and the final mesh block is extracted based on the extent of the requested block.
3. Converting the mesh model to a volumetric one using solid voxelization [46]. This operation is handled with a fast in-house GPU-based voxelization software that uses conservative rasterization [47]. If the input mesh is not watertight, the neuron identifier of the mesh is reported to be fixed.
4. Annotating the volumetric tissue block with the optical properties of the rat brain at the specified region. The optical properties are retrieved from a 3D atlas that was compiled in a recent study by Azimipour et al. [48].
5. Labeling the block with fluorescent dyes to simulate their injection into the intracellular space of the different neurons contained in the generated block. The intrinsic spectroscopic characteristics of the selected dyes are obtained from an online database available at [49].

**Fig. 5:**
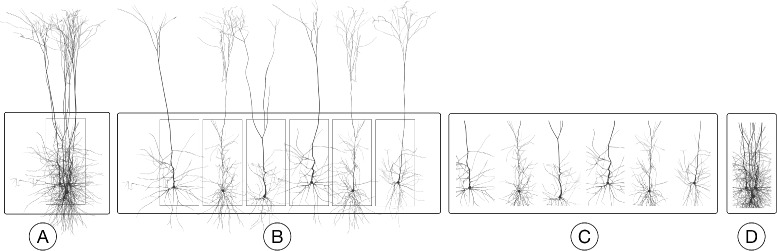
An illustration of the mesh block extraction from the selected targets in the cortical column. **a** The spatial extent of the block is identified by a bounding box that is given in the input configuration. **b** The meshes are generated from the corresponding morphologies with an extended version of the meshing pipeline presented by Lasserre et al. [44]. **c** The resulting wavefront object meshes are loaded in Blender [45] and clipped on a per-mesh basis. **d** All the clipped meshes are loaded in Blender and grouped together with a union boolean operation to generate the final mesh block

In some cases, the experiments are limited to investigate the responses of individual neurons, pair of neurons or a small set of neurons. The generation of a fluorescent tissue block for such experiments is relatively easy as described in the aforementioned process. In contrast, other experiments require extracting a large tissue block that might assemble hundreds or thousands of neurons. The spatial extent of this block does not necessarily enclose the bounding volumes of all the neurons that are located into it because the positions of the neurons are identified based on their cell bodies (or somata). Extracting a tissue block from a large cluster of neurons following the previous approach on a single computing node is inefficient and in some cases is impractical. To resolve this issue, we have developed a parallel workflow that can efficiently generate high density tissue blocks. This workflow runs on high-end visualization clusters that consist of several computing nodes connected together via high bandwidth networking infrastructure. This workflow, shown in Fig. (5), parallelizes the mesh generation and clipping operations exploiting all the available nodes in the cluster.

### Pipeline implementation

Implementing our optical model requires a physically-based spectral rendering framework that can model the light rays by spectral distributions as an alternative to the tri-stimulus representation. The physically-based rendering toolkit (PBRT) [50] has been chosen amongst other systems like Mitsuba [51] or LuxRender [52] due to the existence of an accompanying reference [38] that documents the software architecture of the framework. Though, it only supports CPU-based rendering, which will limit the rendering performance for high resolution images with sufficient sampling densities.

We have implemented our estimator in Eq. (10) in a volumetric integrator class that can be selected in the configuration file given to run the rendering framework. We have also extended the volumetric grid class to support loading annotated fluorescent volumes to allow tagging the same model with multiple fluorescent dyes. The automated block extraction pipeline is configurable to generate PBRT scene description files and render them directly after the creation of the fluorescent tissue block volume.

## Results, validation and discussion

**Fig. 6:**
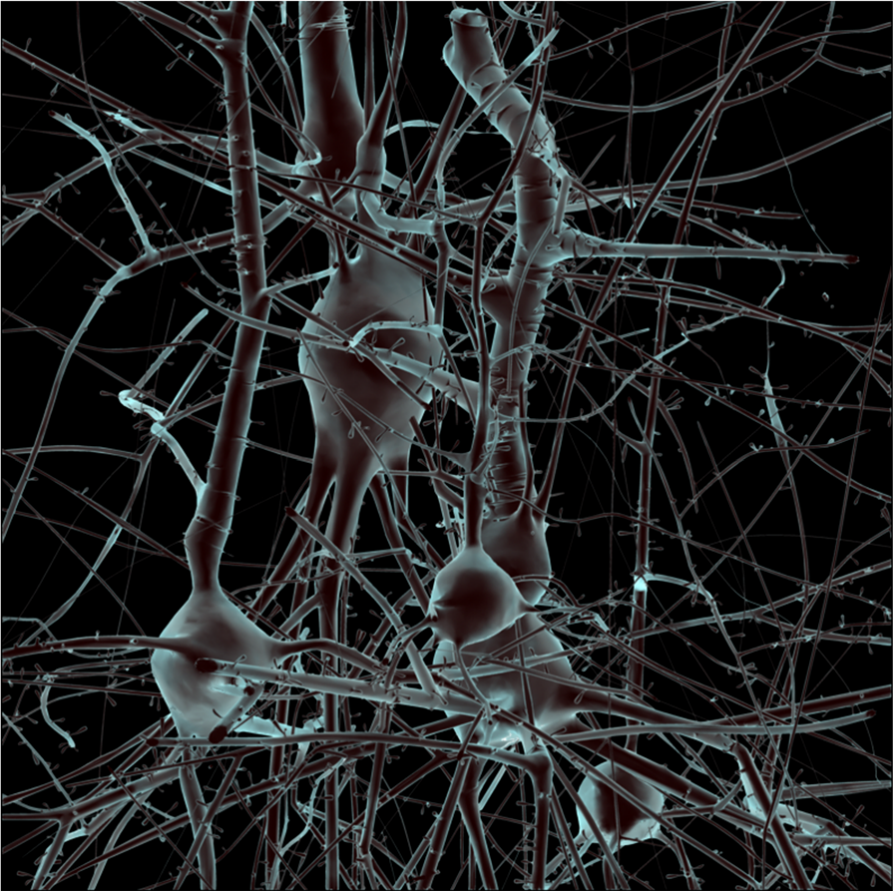
Surface rendering of a watertight mesh of a 50 *μ*m^3^ tissue block extracted from a digital reconstruction of the microcircuitry of the somatosensory cortex of a two-week-old rat. The model is textured with an electron microscopy shader and loaded in Maya (Autodesk, California, USA) [59] for rendering

The results of our visualization pipeline are demonstrated on a 50 *μ*m^3^ tissue block extracted from the center of the neocortical column model (Fig. 4). A surface rendering image of the surface mesh of this block (prior to virtual fluorescent injection) is illustrated in Fig. 6.

From this extracted mesh block, we have created two experimental sets of fluorescent-annotated volume blocks. The first one is tagged with the same type of fluorescent dye dissolved in several solutions having different extinction coefficients. The goal of this set is to experiment the responses of the same fluorescence parameters in the presence of relatively low, medium and high scattering volumes. The other set is labeled with various fluorescent dyes that have different spectral responses at fixed concentrations. This set is designed to validate and measure the performance of our extended optical model that can simulate the light interaction with fluorescent volumes. The two sets were labelled with multiple dyes from the Alexa Fluor family, Alexa Fluor 350, 488, 568 and 633. This family is selected in our experiments due to its importance in fluorescence microscopy and cell biology in general [53]. Table 2 summarizes some of the spectroscopic properties of the four dyes including their maximum excitation and emission wavelengths (nm), molecular weight (kDa), quantum yield, and molar absorptivity (cm^−1^M^−1^).

**Table 2:** The properties of all the fluorescent dyes used to label the tissue model

Dye	Properties					
	Color	MW	*λ*_*x*_	*λ*_*m*_	*ϕ*	*ε*
Alexa F. 350	Blue	410	346	442	0.02	19000
Alexa F. 488	Green	643	495	519	0.92	73000
Alexa F. 568	Orange	792	578	603	0.69	88000
Alexa F. 633	Red	1200	632	647	0.90	159000

**Fig. 7:**
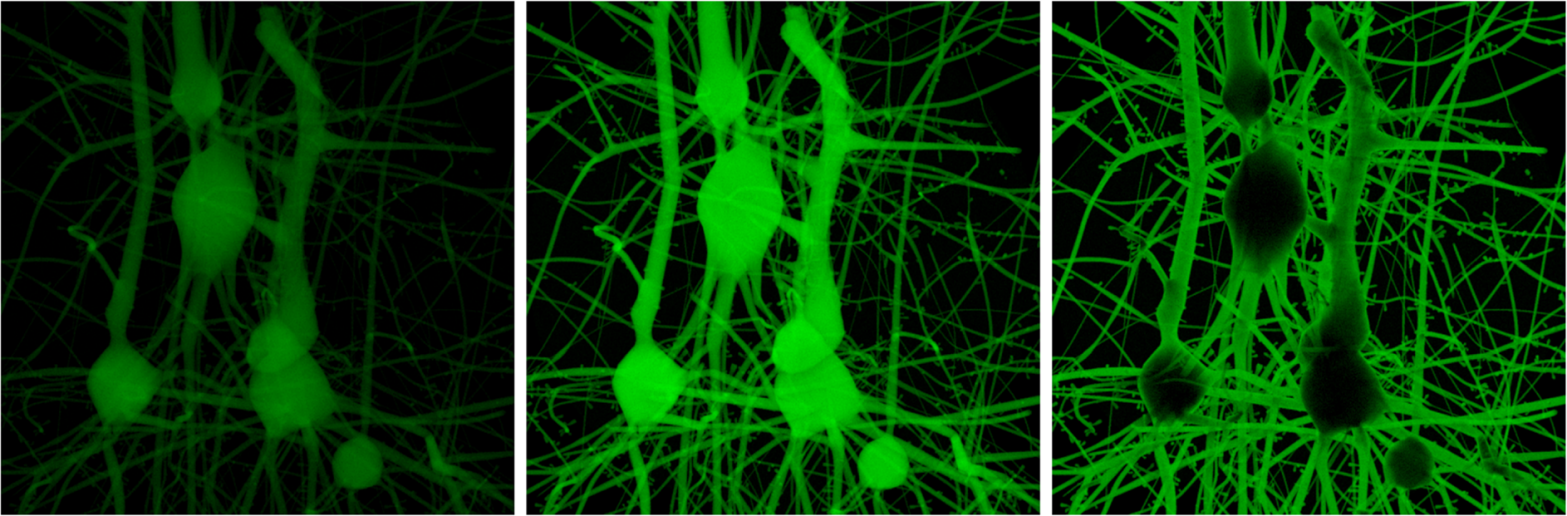
Volume rendering of a 50 *μ* *m* ^3^ fluorescent neuronal model block tagged in silico with three Alexa Fluor 488 solutions that are characterized by low (*left*), medium (*middle*) and high (*right*) extinction coefficients. The volumes are illuminated with monochromatic diffusive light source that emits at 495 nm corresponding to the maximum excitation wavelength of the Alexa Fluor 488 dye

The first set is labelled with three Alexa Fluor 488 solutions that are characterized with extinction coefficients that are 10, 100 and 1000 times greater than that of pure water [54]. To maximize the emission, the illuminating light source is set to emit at the maximum excitation wavelength of Alexa Fluor 488 at 495 nm. Figure 7 shows the results of rendering the three tissue volume blocks under the same illumination conditions.

**Fig. 8:**
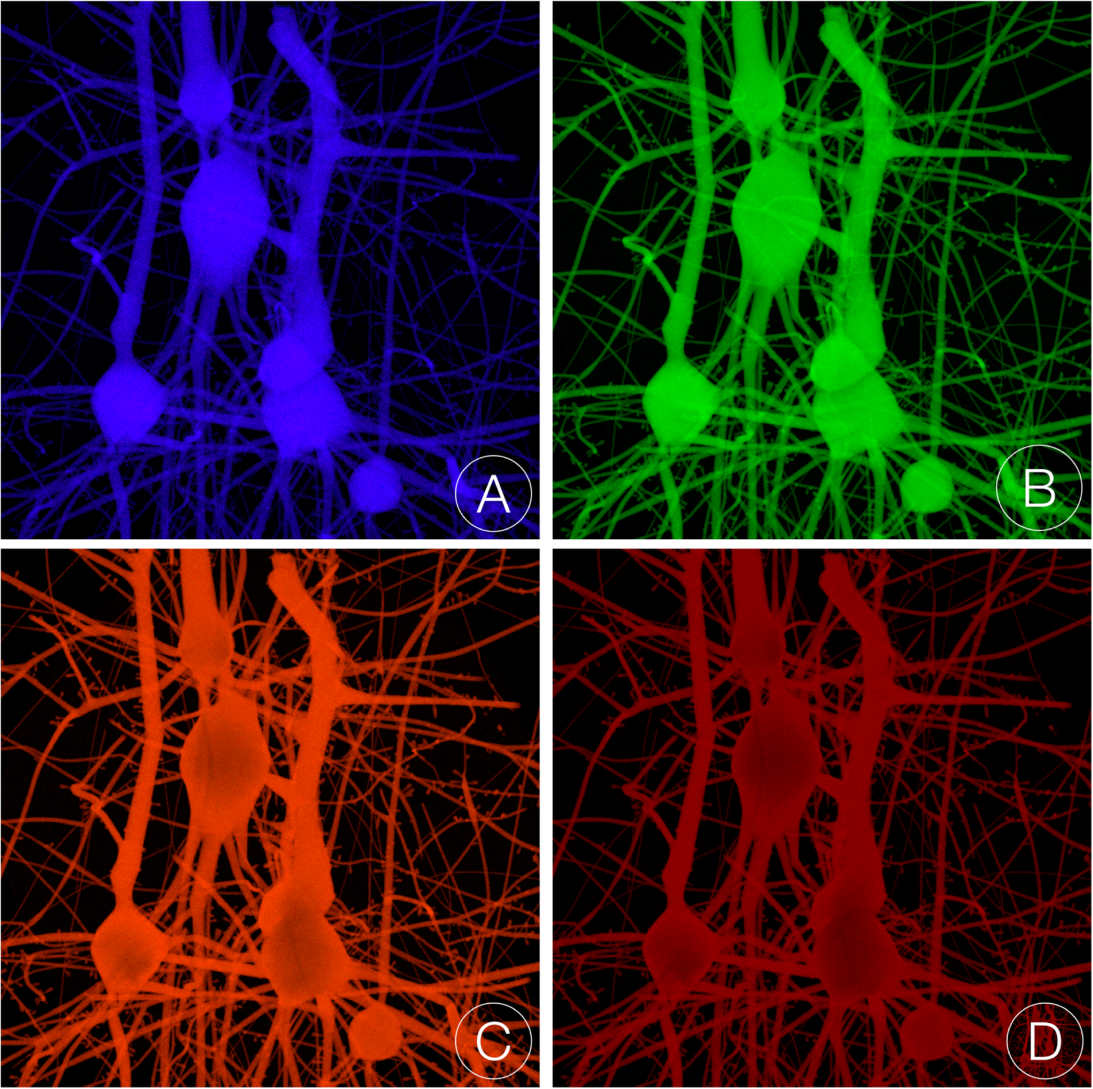
Volume rendering of the tissue volume blocks when the neurons are virtually injected with four different fluorescent dyes: **a** Alexa Fluor 350, **b** Alexa Fluor 488, **c** Alexa Fluor 586 and **d** Alexa Fluor 633. The volumes are illuminated with monochromatic laser sources at 346, 495, 578 and 632 nm that correspond to the maximum excitation wavelength of the four fluorescent dyes respectively

The tissue blocks in the second set are tagged with Alexa Fluor 350, 488, 568 and 633 solutions at the same concentration (0.4 mol/l). The same illumination conditions defined in the first experiment are used to excite the volumes in this case where the light source emits at the maximum excitation response of each respective dye (refer to Table 2). Figure 8 illustrates the images rendered for the four tissue volume blocks used in this experimental set.

### Fluorescence optical model validation

The experimental measurements of the excitation and emission spectra of fluorescent dyes are normally recorded for highly diluted and low scattering solutions using Beer-Lambert law and the fluorescence brightness equation [55]. However, the normalized spectral distributions of the emission spectra recorded from highly scattering solutions should have similar profiles to the experimental emission spectra of the fluorescent dyes [56]. In this context, we validated our fluorescence optical model relying on two basic tests. The first one measures the SPD of the generated images from our visualization pipeline and then compares their normalized profile with the distribution of the intrinsic emission spectra of each dye. Note that the SPDs of each image are recorded before their conversion to RGB colors for each pixel in the image.

**Fig. 9:**
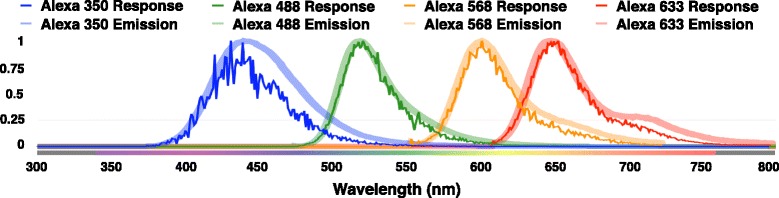
Normalized emission SPDs measured from the images illustrated in Fig. 8. The spectral responses of the emission recorded from each tissue block is qualitatively compared with the actual emission spectra of the four Alexa Fluor dyes used to tag the tissue block. The SPDs are obtained at the maximum excitation wavelengths of each respective dye (346, 495, 578 and 632 nm) and 1024 spectral samples per pixel

The four tissue volume blocks in the second experimental set are used to validate our optical model. The normalized spectral responses (or SPDs) from the four images shown in Fig. 8 are compared to the emission profiles of the four dyes. The results of this validation test are shown in Fig. 9.

**Fig. 10:**
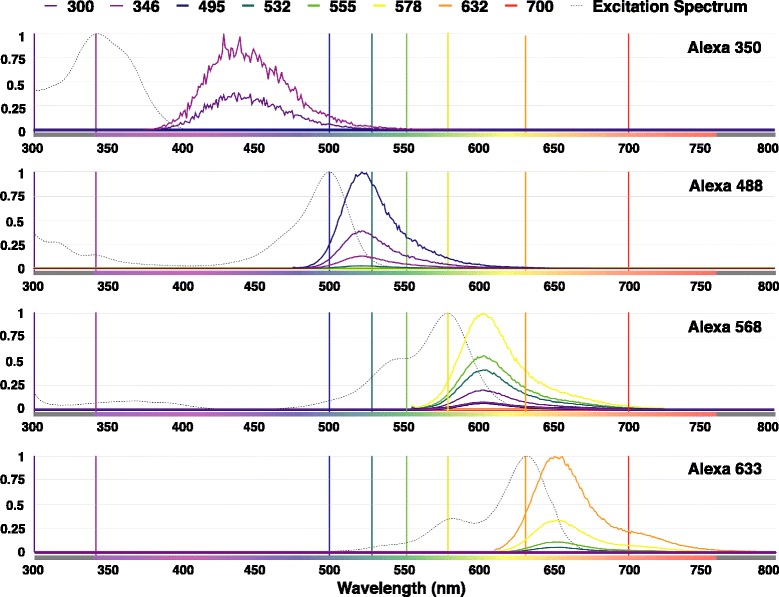
Relative emission SPDs measured from the images generated from rendering the four fluorescent tissue blocks tagged with Alexa Fluor 350, – 488, – 568 and – 633 at different excitation wavelengths between 300 and 700 nm. The profiles are normalized to the SPD measured at maximum excitation wavelength for each respective dye. The SPDs are detected at 1024 spectral samples per pixel. Notice the relation between the amplitude of the excitation spectrum of each dye at the exciting wavelength and maximum amplitude of measured SPD

The second validation test measures the performance of the model when the volume is illuminated with different wavelengths. Depending on the excitation spectrum of the dye and the selected wavelength to illuminate the solution, the scale of the emission spectrum is proportional to the amplitude of the excitation spectrum at the excitation wavelength. The maximum emission profile is reached when the maximum excitation wavelength is used [55, 57]. In this test, all the tissue volume blocks are illuminated at several wavelengths (300, 346, 495, 532, 555, 578, 632 and 700 nm) and the responses are recorded and relatively compared. The results of this test are illustrated in Fig. 10.

### Pipeline evaluation

The rendering results were evaluated collaboratively with a group of different experts in neurobiology and in silico neuroscience. They all agree that the renderings are similar to what they visualize under the microscope. They were also excited to see how the responses are changing when the optical and spectroscopic properties of the dyes are varied. This would allow them characterizing the responses of the neurons in various regions of the brain that have different optical properties. The scientists working in the brain simulation team have expressed their interest in applying our pipeline to their data to validate their in silico VSDI experiments against realistic data recorded by the fluorescence microscope. Other scientists have requested further extensions of the pipeline to visualize neuroglial cells.

### Rendering performance

In general, Monte Carlo rendering is known to be time consuming. The rendering performance of Monte Carlo-based algorithms depends on multiple factors including the pixel sampling density, number of light samples, optical properties of the volume and the image resolution as well. If the sampling rates are relatively low, the rendered image will be full of noise. Therefore, high sampling is mandatory to have an image with a converging solution. Our results have been rendered with pixel sampling of 512×512 samples per pixel. Moreover, high spectral sampling is also required to obtain accurate emission spectra that can reflect those measured in real spectroscopic experiments. We have used a spectral sampling of 1 nm. The rendering time of the images demonstrated in Figs. 7 and 8 varied between six and eight hours on a recent machine that is shipped with Intel core i7 CPU and 32 GBytes of memory.

## Conclusion and future work

**Fig. 11:**
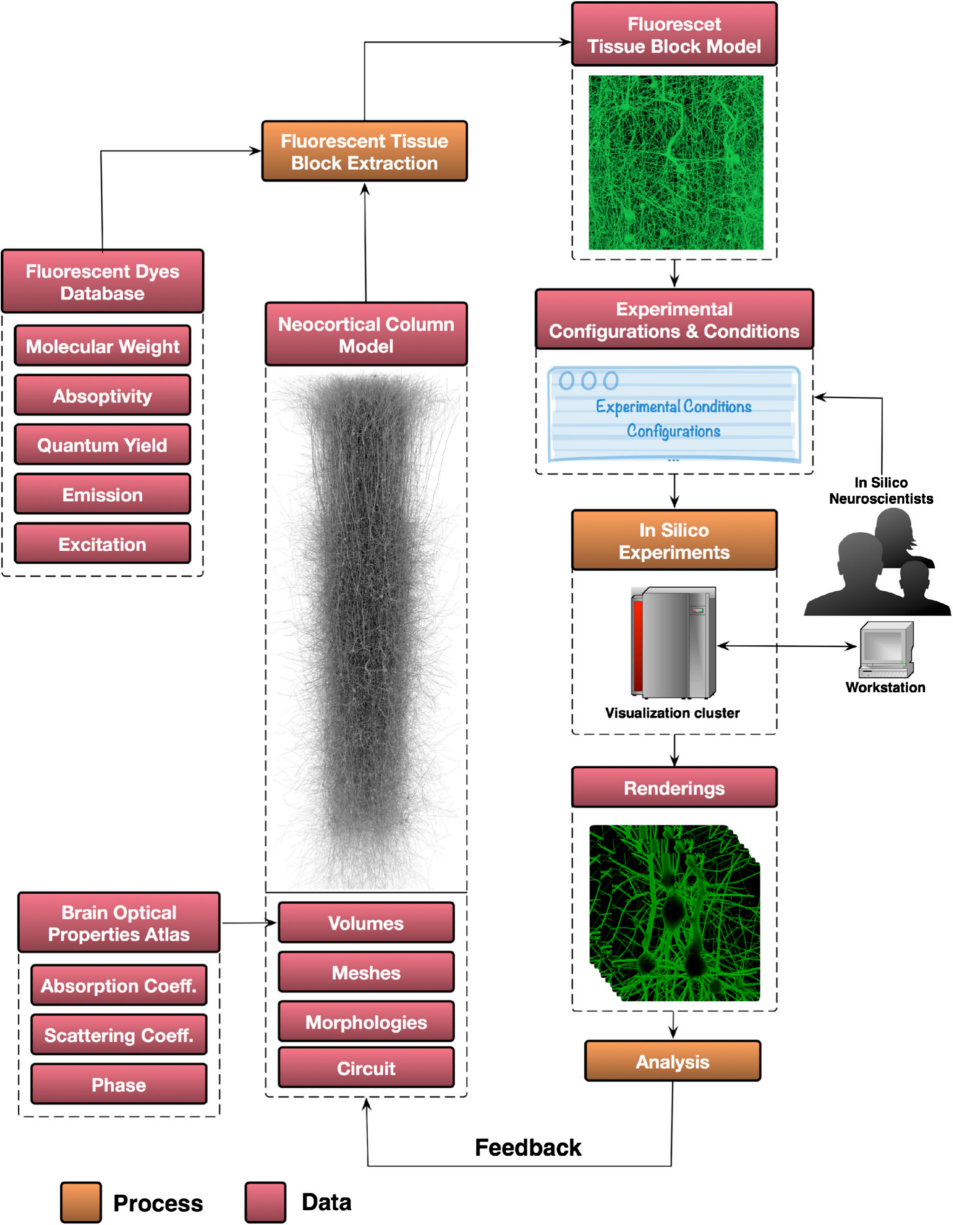
A high level overview of the in silico experimentation workflow. The scientists extract a tissue block from the neocortical column model, tag it virtually with a specific fluorescent dye and use it in in silico fluorescent-based experiment. The renderings are analyzed and validated, and the tissue model is improved

The current visualization systems are limited to meet the immense challenges of in silico neuroscience, where biological experimentation are performed in computer simulations. A wide range of those experimental observations rely on fluorescence imaging to reveal several structural and functional aspects of the brain. Reproducing the same experimental procedures in silico is subject to the existence of visualization engines that can handle fluorescent models. We presented a visualization pipeline to address these challenges. The pipeline is composed of a generic volume rendering system capable of visualizing highly scattering fluorescent volumetric datasets. This system is applied to visualize virtually-tagged fluorescent tissue blocks that are extracted from a unifying model of the neocortical microcircuitry reconstructed from rats. The pipeline is primarily developed to assist the neuroscientists exploring and analysing their in silico experimentations that incorporate those fluorescent blocks to present a visual feedback that allows them fine tuning their experimental parameters and improving the model in an iterative manner (Fig. 11).

A rigorous bio-physically-based optical model is developed to account for light interaction with highly scattering fluorescent media. This model accounts for the optical properties of the tissue and also the spectroscopic properties of fluorescent dyes. The model is qualitatively validated against the the profiles of the spectra of multiple synthetic fluorescent dyes.

We are currently extending this pipeline to visualize the simulation data of in silico VSDI experiments to validate the simulation of the cortical activity for a large meso-scale circuit and also to visualize neuroglial cells. We are also working on accelerating the performance of the rendering workflow by providing a high performance distributed solution on multi-GPU visualization clusters based on the framework presented by Eilemann et al. [58].

## Abbreviations

3D: Three-dimensional
CPU: Central processing unit
GPU: Graphics processing unit
PDF: Probability density function
PBRT: Physically-based rendering toolkit
RTE: Radiative transfer equation
SPD: Spectral power distribution
SFP: Simulated fluorescence process
VSDI: Voltage sensitive dye imaging
